# Dental Department of the University of California

**Published:** 1883-02

**Authors:** 


### EDITORIAL.

### Dental Department of the University of California.

The Dental Department of the University of California, held its *first* commencement exercises at Binai Brith Hall, San Francisco, on Wednesday evening, November 8, 1882.

An address on behalf of the State, was delivered by Governor

George C. Perkins, and also one on behalf of the Board of Regents by Rev. Horatio Stebbins, A.M., D.D.

The Valedictory on behalf of the Faculty was delivered by the Dean, Prof. S. W. Dennis, M.D., D.D.S., and that on behalf of the graduating class by Henry John Plomteaux.

The number of matriculates was thirty-two.

The degree of D.D.S. was conferred on the following members of the graduating class by W. T. Reid, A. M., President of the University :—Thomas Watson Hall, Oakland, California; Charles Wesley Hibbard and Thomas Morffew, San Francisco, Cal.; Henry John Plomteaux, Oakland, California; Charles Wesley Richards, William Harry Stanley, Gustav William Sichel, M. D., August VanCrombrugghe, San Francisco, California.

N. B.—Unlike the Eastern colleges, the Medical and Dental Departments of the University of California, hold their sessions in summer instead of in winter.

